# Recognition of physiotherapists’ expertise in Parkinson’s disease

**DOI:** 10.1186/1472-6963-13-430

**Published:** 2013-10-23

**Authors:** Nicole ABM Ketelaar, Marten Munneke, Bastiaan R Bloem, Gert P Westert, Marjan J Faber

**Affiliations:** 1 Scientific Institute for Quality of Healthcare, Radboud University Nijmegen Medical Centre, PO Box 9101, IQ healthcare 137, 6500 HB, Nijmegen, The Netherlands; 2 Department of Neurology, Donders Institute for Brain, Cognition and Behaviour, Radboud University Nijmegen Medical Centre, Nijmegen, The Netherlands

**Keywords:** Provider choice, Comparative performance information, Physiotherapy, Expertise, Parkinson’s disease

## Abstract

**Background:**

Publicly available information comparing performance across quality and costs has proliferated in recent years, both about individual healthcare professionals and hospitals. This type of information is now becoming increasingly available for physiotherapists with expertise in Parkinson’s disease (PD). Our study aimed to explore the ability of people with Parkinson’s disease to recognise expertise, and to what extent respondents selectively choose such expert physiotherapists.

**Methods:**

We used claim data from the period 2009–2010 to select customers with PD who claimed physiotherapy. A random sample of 500 eligible respondents received a paper-based survey. We used descriptive statistics to compare the respondent characteristics, a qualitative programme to analyse the qualitative items, and univariate and multivariate regression.

**Results:**

Most respondents (89%) took their referring physician’s advice when selecting a physiotherapist, although this advice rarely was supported with arguments. The remaining respondents (11%) searched for comparative performance information about physiotherapists. Respondents who recognised the added value of PD expertise among physiotherapists were 3.28 times as likely to search for comparative performance information as those who did not understand. Respondents were willing to switch to an expert physiotherapist (68%), and this willingness increased if they recognised the value of PD expertise (*p* < .001).

**Conclusion:**

The participants were able to recognise certain aspects of expertise. Though they showed relatively few signs of selectively choice behaviour for expert physiotherapists. Both respondents and referring professionals need more understanding about the added value of an expert physiotherapist, to foster selective provider choice.

## Background

The release of information in the public domain about the performance of healthcare providers has become a strategy for improving the quality of healthcare [1]. Providing such comparative performance information (CPI) may empower and enable patients to identify and choose high-quality providers [2,3]. While we have an increasing understanding of motivating factors for selective provider choice regarding elective conditions [4-6], or primary care physicians [7] there is no knowledge available about whether this evidence also applies to people with a chronic disorder such as Parkinson’s disease (PD). Our aim is to explore whether people are aware of, and able to recognise expertise, as an expression of quality of care, in PD among physiotherapists.

There are an estimated 1.2 million people with Parkinson’s disease (PwP) in Europe [8]. Physiotherapy is part of the treatment for many PwP [9]. In the Netherlands and the United Kingdom, 54–60% of all PwP receive physiotherapy [10,11]. In the former, physiotherapy is mainly provided in community-based settings, in provider-owned practices. The reimbursement of the first 21 physiotherapy sessions, on annual basis, depends on the consumers additional insurance package. Though, for the treatment of PD there is an unlimited reimbursement out of the basic insurance after these twenty-one treatments. Dutch patients have free access to physiotherapy since 2006 so they can self-refer to a physiotherapist, which is considered to be a structure premise for selective provider choice [12].

More than 75% of the Dutch allied health professionals report a lack of PD-specific expertise. More than half of them were unfamiliar with the treatment options of other professionals, and they had not participated in relevant educational programmes [9]. In response to this gap of knowledge, a Dutch multidisciplinary network, ParkinsonNet, was developed and implemented to increase the PD expertise of physiotherapists [13]. ParkinsonNet is a multifaceted intervention that includes several elements (Table 1).

**Table 1:** **Five core elements of ParkinsonNet [**[14]**]**

	
1	Delivering care according to evidence-based guidelines
2	Continuous education and training of therapists (including physiotherapists, occupational therapists, and speech therapists)
3	Structured and preferred referral to ParkinsonNet therapists by neurologists, enabling each therapist to attract a large volume of patients to increase expertise
4	Encouraging communication and regional collaboration with referring physicians
5	Promoting visibility of the available expertise for both patients and professionals

Launching of the sixty-fifth regional ParkinsonNet-network in 2010, national coverage was achieved. Currently, about 2700 professionals are connected throughout the Netherlands [14]. Although, physiotherapists can be expert in PD without being connected to this multidisciplinary network, the term 'expert physiotherapist’ is exclusively used throughout this paper for physiotherapists who are connected to ParkinsonNet. An expert physiotherapist received additional training in treating PwP. Evidence shows that specific treatment options provided by expert physiotherapists are more beneficial to patients [15,16]. Also, expert physiotherapists show better guideline adherence scores compared to generically active physiotherapists and their PwP volume is larger [9].

Awareness of referral options for other professional disciplines [17] and knowledge and use of the Dutch PD guideline [13] are necessary to attain expertise in PD. The implementation of ParkinsonNet increased the number of PwP treated by each physiotherapist [18]. However, approximately 70% of the PwP are still treated by a general physiotherapist [18]. This raises the questions whether PwP are aware of PD expertise in physiotherapy and whether they understand its value, i.e. these are, apart from free provider choice, two additional relevant conditions for selectively choosing a provider.

This paper focuses on ability of PwP to recognise expertise in PD among physiotherapists, as a potential indicator of selective provider choice. We consider this in several ways: by seeking the perspective of PwP about the PD expertise of their physiotherapist, by ascertaining the descriptions of PD expertise from the perspective of PwP in a qualitative way, and whether physiotherapists’ PD-related expertise influences the search for CPI among PwP. In addition, we examine the role of referring physicians. We take into account factors that might affect provider selection such as consumer characteristics (e.g. age, education, and internet use), knowing where to search for CPI, the willingness to switch, and respondents’ expectations regarding variation in quality of care.

## Methods

### Design and study population

Our paper-based survey focused on the selective provider choice for expert physiotherapists in a cohort of people with PD. We selected PwP on the basis of claim data in the period 2009–2010 of a Dutch healthcare insurance company that accounts for 20% of the Dutch insurance market. We approached eligible candidates, that is, people who were registered as having PD in the diagnosis–treatment combination (Dutch version of diagnosis related groups) combined with an episode of physiotherapy in the year prior to our survey. Only consumers who received physiotherapy treatment for PD were included because we wanted to focus on peoples’ capacity to recognise physiotherapists’ PD expertise. People who had had physiotherapy for PD for several years were excluded; the decision-making process (deliberate or not) had to be recent to exclude recall bias. A total of 886 patients met these eligibility criteria. We sent surveys to 500 participants randomly selected from this group. Due to privacy legalities, the insurance company drew the sample. According to local regulations in the Netherlands (Commission involving human subjects research) (CMO) region Arnhem-Nijmegen) this study did not need approval of the ethical review board.

### Measures and data collection

The survey consisted of 37 items. The first items ascertained the treatment with physiotherapy for PD in the past year to satisfy our inclusion criteria. We used five items to operationalise the recognition of physiotherapists’ PD expertise. Two items explored the level of awareness and the descriptions of PD expertise from the perspective of the participants: awareness of the existence of expert PD physiotherapists (dichotomous variable), and participants were asked to estimate whether their physiotherapist was an expert in PD. This latter item was answered on a three-point scale: yes, no, or do not know. We compared the participants’ views of physiotherapists’ expertise on the basis of claim data with the ParkinsonNet data to see if they correlated. The respondents answered an open-ended question that dealt with their assessment of physiotherapists’ expertise. We categorised the answers into eight core themes based on a framework for patient centeredness in PD [19] and quality of care domains as formulated by the Institute of Medicine [20]. Result section, Recognition of physiotherapist expertise in Parkinson disease). We estimated the search for CPI by addressing the last two aspects used to operationalise the recognition of physiotherapists’ PD expertise: recognition of the added value of PD expertise, and whether participants paid attention to PD expertise among physiotherapists when they selected a provider (all dichotomous variables).

Knowing where to look for CPI was a dichotomous variable. Expectations of variation in the quality of care between a generic physiotherapist and an expert physiotherapist formed a categorical variable, determined on a four-point scale: yes, large differences; yes, small differences; do not know; or no differences. The willingness to switch to an expert physiotherapist was determined on a five-point scale: most likely, likely, unlikely, most unlikely, and do not know. We dichotomised this into likely (most likely, likely) versus unlikely (most unlikely, unlikely, do not know) because the distribution of this variable was positively skewed.

We assessed the role of referring physicians with the following questions: do referring physicians provide you multiple choice options for physiotherapists about where you could go to? Did your referring physicians give you an advice to which provider you should go for the *best* treatment? If so, is this advice accompanied by arguments and by CPI, and do you take this advice? (All of these are dichotomous variables).

We asked the participants which attributes they searched for when choosing a physiotherapist, and for which of these attributes referring providers supplied them with information. The attributes contain items about services and quality of care. The first three items were available for PwP at the time the survey was send out, we added items 4 and 5 because we expected that these items soon become available as well and/or these items were common items with other (elective) conditions.

1. ParkinsonNet membership, e.g. practices connected to this network.

2. Information regarding the added value of being treated by an expert PD physiotherapist.

3. Distances to physiotherapy practices.

4. Physiotherapists with PD expertise.

5. Experiences of PwP who were treated by an expert physiotherapist.

We also ascertained the demographics of the study participants. We treated age as a continuous variable. The variable of educational level was described as none/low, average, or high. We used the stages defined by Hoehn and Yahr [21] to describe the self-reported disease characteristics. The Hoehn and Yahr (HY) stages range from no PD signs in stage 0 to needing a wheelchair or being bedridden in the most severe stage 5. Patients’ disease severity was classed as mild (HY stages 0–1), moderate (HY stages 2–3), or severe PD (HY stages 4–5).

The survey was field tested and optimised for ten patients and four PD researchers. The final survey was sent by post, and a reminder was posted 2 weeks later. Data were collected in October and November 2010.

### Analysis

The survey contained four qualitative questions. The filled-out paper-copy surveys were scanned and transcribed into an electronic format, by means of an automated process (Teleform). We did not use a separate programme for the qualitative analysis, such as Atlas.ti, as it did not bring much benefit for the rather small amount of qualitative data. Though we did apply the principles of thematic analysis (conducted by NK and MF), supported by the PD-specific framework for patient centeredness [19] and a general framework for quality of care [20]. Descriptive statistics were calculated for each survey item and compared with respondent characteristics. We explored the association between how patients value PD expertise and the search for CPI (treated as a dependent variable determined by 'yes’ or 'no’). We used univariate logistic regression analysis to separately examine the associations between the independent variables and the search for CPI. The independent variables were: age, education, internet use, awareness of the existence of expert PD physiotherapists, an understanding of the added value of an expert PD physiotherapist, prior attention to physiotherapists’ expertise, knowing where to search, expected quality differences between generic physiotherapists and expert physiotherapists, willingness to switch, and the provider options named by referring physicians.

Statistically significant variables (*p* < 0.05) were included in the stepwise forward multivariate logistic regression analysis. We calculated the outcomes separately for each independent variable while controlling for the other variables in the model. We presented these outcomes with odds ratios (ORs) and 95% confidence intervals (CIs). We used SPSS 18.0 to carry out the analyses.

## Results

### Demographics

In total, 380 respondents completed the survey (gross response rate = 76%), and 320 surveys were analysed (net response rate = 64%). The 60 participants whose surveys were excluded had not received physiotherapy for PD, but for another medical condition. Table 2 presents the background characteristics of the study population.

**Table 2:** Background characteristics of study population

**General characteristics**		***n***	**%**
Gender	Male	147	56
Mean age in years ± SD		72	±9
Level of education			
	None/low	165	56
	Average	91	31
	High	39	13
Residential status			
	Alone	55	18
	Together	211	71
	Other situation	36	11
Internet use	Yes	85	28
**Specific Parkinson’s Disease characteristics**			
Diagnosis			
	Parkinson’s disease	292	95
	Atypical parkinsonism	10	3
	Unknown	4	2
Hoehn and Yahr stage (self-reported)			
	Mild (0–1)	83	30
	Moderate (2–3)	127	41
	Severe (4–5)	87	29
Use of physiotherapy in the past year	Yes	320	100

Because of missing data in the background characteristics, not every score accumulates to the total of 320.

### Quality of care for Parkinson’s disease

Sixty percent of the study population expected quality differences in the care provided by generic physiotherapists and expert physiotherapists, and 34% of them expected these variations would be large. A minority of participants (5%) expected no quality differences, and the remaining respondents (35%) said they did not know. Of those who expected quality differences, 33% did not know what kind of value an expert physiotherapist could add. In total, about half the study population (51%) did not know the added value of an expert physiotherapist. Participants who expected to find quality differences were younger than participants who did not expect quality differences (71 ± 10 years versus 74 ± 8 years, *p* = .009).

More than two-thirds of the respondents (68%) were willing to switch to an expert physiotherapist if it turned out that their current physiotherapist had no PD expertise. The distance the participants were willing to travel to see an expert physiotherapist was 5 km (interquartile range: 2–11 km). Respondents who had previously heard about expert physiotherapists were more willing to switch (82% versus 54%, *p* < .001) and respondents expecting differences in the quality of care were also more likely to switch (87% versus 31%; *p* < .001).

### Recognition of physiotherapist expertise in Parkinson disease

Most participants (74%) had already heard about expert physiotherapists. Fewer participants (46%) said they had previously paid attention to whether the physiotherapist was an expert in PD before selecting a physiotherapist. Participants who had previously heard about physiotherapists with PD expertise had a higher educational level than those who did not know about expert physiotherapists (*p* = .001). Awareness of expert physiotherapists was also related to age. Participants who were aware of expert physiotherapists were younger than those who were unaware (71 ± 10 years versus 76 ± 8 years, *p* = .001).

We asked those who had already heard about expert physiotherapists whether they were treated by an expert PD physiotherapist (*n* = 229). More than 70% asserted they were treated by an expert, 12% stated that they were not, and 17% said they did not know. A comparison of the answers of our study population with ParkinsonNet data showed that 28% of our respondents were being treated by a ParkinsonNet-affiliated physiotherapist.

Participants reported various themes describing what physiotherapists’ expertise and knowledge stands for. Table 3 presents the core themes and the underlying descriptions that the respondents gave. Thirty percent of the respondents said that physiotherapists’ treatment, exercises, and information express a degree of expertise.

**Table 3:** Themes related to the perceived expertise of physiotherapists from the patients’ perspective

		***n***
**Treatment and therapy**		69
Treatment and exercises	*'Expertise is related to the good exercises I have to do at home’* (Woman, 82 years old)	
	*'Right exercises for posture and movement’* (Man, 76 years old)	
**Tailored information and communication**		66
Information about the disease, (practical) advice, counselling, and communication with physiotherapist	*'All arguments with the given instructions are correct’ (*Man, 64 years old)	
	*'He tries to find matching exercises for me in person’* (Man, 70 years old)	
	*'She knows a lot of Parkinson Disease, I receive good answers to my Parkinson Disease related questions*’ (Woman, 74 years old)	
**Knowledge of Parkinson disease**		34
Knowledge about and expertise in Parkinson disease symptoms. Noticing changes of condition	*'He knows exactly what to do’* (Man, 78 years old)	
	*'He knows the Parkinson disease signs and tries the recommended treatment’* (Man, 57 years old)	
	*'She knows the limitations Parkinson disease imposes on me, and gives useful suggestions’* (Woman, 82 years old*)*	
	*'Works with several patients who have Parkinson disease’* (Man, 60 years old)	
**Effectiveness**		25
Improved condition, increased mobility, decreased freezing	*'I can to move better and my muscles are less stiff since the treatment’* (Woman, 82 years old)	
	*'My condition has improved since this treatment’* (Man, 75 years old)	
**Cooperation with caregivers**		19
Need for interdisciplinary care. Working within a network with allied healthcare providers	*'He works with the neurologist’* (Man, 73 years old)	
	*'He is involved in the Park fit studies’* (Man, 71 years old)	
**Emotional support, empathy, and respect**		16
The patient receives interest and attention, is taken seriously, patient’s disease is accepted and coped with	*'Shows great interest’* (Man, 74 years old)	
	*'Shows interest, takes me seriously’* (Woman, 77 years old)	
	*'Is very patient’* (Man, 88 years old)	
**Unable to define the expertise of the physiotherapist**		4
Difficult to access the providers’ expertise	*'I do not know which exercises are the best for patients with Parkinson disease’* (Man, 61 years old)	
**Treatment evaluation**		2
Providers ask questions about the treatment	*'Takes the time to evaluate the treatment process’* (Man, 70 years old)	
**Accessibility of healthcare**		2
Treatment or physiotherapy at home	*'Treats me at home’* (Man, 70 years old)	
Total		103

### The role of referring physicians

The respondents were referred by: neurologists (49%), general practitioners (GPs; 18%), and specialist Parkinson’s nurses (18%). Another 15% of the respondents saw a physiotherapist on their own initiative. About half the participants who received a referral were provided with additional information regarding expert PD physiotherapists. Only a minority of the respondents (25%) received multiple choice options for a physiotherapist. Although 85% of the participants found it important to choose their own physiotherapist, most (89%) of those with a physician referral took their physician’s advice when selecting a physiotherapist.

### The search for comparative performance information

Most respondents (89%) reported not having searched for CPI when it became clear they needed physiotherapy. Some respondents gave more than one reason for not searching. The most important reason was not perceiving any need for more information (60%). Other reasons were: no internet access at home (29%), not knowing where to search (15%), not knowing how to search (9%), lack of motivation (13%), not knowing how to look for information (12%). A smaller group found that more information led to more doubt (7%), some felt that it was too much responsibility (4%), and some had no time (3%).

Participants who searched for information (11%) wanted to know about: practices connected to ParkinsonNet (*n* = 13, 4%), expert PD physiotherapists (*n* = 18, 6%), physiotherapist practices close to home (*n* = 19, 6%), experiences of patients who received treatment from an expert physiotherapist (*n* = 7, 2%), and what added value physical treatment from an expert physiotherapist can give a person with PD (*n* = 11, 3%). Some declared they did not find the information they would have like to have. Others reported that the information was too general and not trustworthy.

Univariate logistic regression analyses revealed that several variables are associated with the search for CPI. Respondents’ awareness of expert PD physiotherapists, an understanding of the added value of an expert physiotherapist, and the willingness to switch to an expert PD physiotherapist were statistically significant, as were consumer characteristics (age and internet use). These variables were included in the multivariate regression analysis.

The stepwise multivariate logistic regression analysis revealed that recognising of the added value of an expert PD physiotherapist was the most important predictor for the search for physiotherapists’ CPI (Table 4). The likelihood of people who recognised the added value searching for information was 3.28 times as great as the likelihood for those who did not recognise (OR = 3.28 [95% CI 1.42–7.58]).

**Table 4:** Univariate and forward stepwise multivariate regression relationship of searching for comparative performance information versus background characteristics, awareness, and understanding physiotherapists’ expertise in Parkinson’s disease

	**Univariate analyses**		**Multivariate analyses**	
	**OR [95% CI]**	***P***	**OR [95% CI]**	***P***
Age	0.96 [0.93–0.99]	0.02*		
Educational level (low)^†^				
Middle	1.49 [0.69–3.23]	0.32		
High	0.73 [0.20–2.61]	0.62		
No internet use	2.12 [1.02–4.39]	0.04*		
Not aware of expert PD physiotherapists	3.76 [1.11–12.70]	0.03*		
No recognition of added value of expert physiotherapist	3.29 [1.47–7.31]	0.01*	3.28 [1.42–7.58]	0.01*
No prior attention to physiotherapist expertise	0.57 [0.18–1.82]	0.34		
Not knowing where to look for CPI	1.13 [0.30–4.16]	0.86		
No quality differences between generic and expert physiotherapists^†^				
Yes, large differences	1.41 [0.29–6.80]	0.67		
Yes, small differences	0.91 [0.17–4.73]	0.92		
Do not know	0.26 [0.04–1.56]	0.14		
Not willing to switch	3.86 [0.89–16.78]	0.07		
Received provider options from referring physician	1.79 [0.77–4.14]	0.17		

The total number of respondents included in the analysis was 279.

*p < 0.05.

^†^Reference category.

CI, Confidence interval; CPI, comparative performance information; OR, odds ratio; PD, Parkinson’s Disease.

## Discussion

The ultimate goal of releasing CPI about the quality of expert physiotherapists is to improve quality of care for people with PD. Consumers of health care have the power to make a contribution to quality of care in competitive health care system by selective provider choice. This study shows that PwP identify aspects of expertise that appeared to align with the IOM-framework for quality of care, and mostly with patient centeredness. Moreover, the majority of participants (74%) were aware of expert and non-expert PD physiotherapists. Participants were able to describe what the PD-specific expertise and knowledge of their physiotherapist means to them. However, we found little evidence suggesting that the influence on how patients value expertise among physiotherapists influences the search for CPI and selective provider choice. Recognition of the additional value of a PD physiotherapist was a strong predictor of such a CPI search. Yet, about half the patients (51%) had this understanding; therefore, this situation can be improved. Our study shows that PwP hardly ever selectively chose a physiotherapist with PD expertise. Most took the physicians’ referral advice (89%), and the influence of CPI in the decision-making process was limited because only a minority searched for such information (11%).

In terms of the way forward, we first discuss the selective referral behaviour of physicians. Currently, very few physicians’ selective referrals to expert physiotherapists occur. Only half the participants were given additional information regarding expert PD physiotherapists. A King’s Fund publication [22] shows that most GPs in the United Kingdom did not give information about their referrals either. Knowing and recognising PD expertise are necessary conditions for providers’ selective referrals. Without these conditions, it is difficult to provide patients with information. It is also important that referring physicians (e.g. neurologists and GPs) proactively recall this knowledge when they advise and refer patients to a physiotherapist. Referring physicians should support consumers choose selectively by discussing their referral options, so that the choice becomes a matter of shared decision-making.

Second, we focus on the consumer’s selective choice behaviour. Previous studies suggest that, although consumers value quality information [23,24], the use of CPI is limited [1,25,26] among different populations and for a diversity of conditions. Our study confirms this discrepancy. The respondents valued free provider choice as important, but usually followed the referring provider’s advice and did not use CPI to choose their physiotherapist. Previous research shows that once people understand the concept of quality of care, they give a higher value to the measures of quality performance [27]. Further research should address in what way this discrepancy can be countered, for example by improving the circumstances so that consumers’ intentions and their behaviour coincide.

Characteristics of expertise, as they were perceived and expressed by PwP in their own words (Table 3), are connected with the definition of patient-centred care for PD [19]. Other quality criteria, like a large PwP volume, having followed specific training in treating PD and a connection to an expert network, which is important for the members of ParkinsonNet, were less frequently mentioned. We therefore conclude that the definition of expertise among respondents was rather narrow. The aspects that were less frequently mentioned by respondents, might need more attention since recent evidence shows that PwP allocated to multidisciplinary PD care have better quality of life, better motor scores, and less depression [28].

Moreover, at a time when cost control dominates the health policy agenda, it is more urgent than ever to support consumers become a force for improving the quality of healthcare [29]. Since the concept of ParkinsonNet is cost-effective guiding patients to physiotherapists that are connected to ParkinsonNet, might contribute to the containment of costs in our health care system. In regions where ParkinsonNet was active, PwP received more physical therapy, there were fewer admissions to nursing homes, fewer people needed revalidation treatment, and the reimbursed costs were lower [30]. These are all reasons for supporting initiatives that enhance knowledge among PwP about the added value of expert physiotherapists, by means of CPI that covers a broad definition of expertise and a range of quality of care criteria.

Twenty-eight percent of our study population received treatment from ParkinsonNet- affiliated physiotherapists, while 70% claimed treatment from expert physiotherapists. This percentage of 28 is in line with previous evidence [18]. Physiotherapists can be expert in PD without being connected to the multidisciplinary network, but the chance of being treated by an expert outside the network is smaller. Moreover, the multidisciplinary network has been implemented across the entire country. It is most likely that respondents overestimate their physiotherapists’ expertise in PD, otherwise they would have to admit receiving treatment from a physiotherapist who might not provide them with the best possible healthcare. Their overestimation makes switching to a physiotherapist with PD knowledge unlikely, as in their view, physiotherapists are already experts in PD. More consumer knowledge and recognition of expert physiotherapists might lead to an adjustment of consumers’ views towards the level of PD expertise among their physiotherapists and eventually to a search of CPI.

### Implications

Demonstrating the value of expert PD physiotherapists to referring physicians is necessary. Further research should explore whether this will encourage selective referral behaviour. In terms of patient participation, more attention is required to clarify physicians’ decisions to refer to an expert physiotherapist.

A practical implication is that CPI should be extended and made more accessible for PwP and their informal caregivers (family and friends). For example by spreading the information through the patient organisations for PwP on their website or magazine, or flyers in the waiting room of primary care practices. Further research is needed to explore how to encourage consumers to use the information. The CPI should also emphasise in more detail how and in what ways expert PD physiotherapists distinguish themselves from generic physiotherapists. Both the added value of expert physiotherapy and the multidisciplinary element should be emphasised.

### Limitations

This study is not without shortcomings. First, as the understanding of the added value of an expert physiotherapist is related to the search for CPI, it would have been better to have more detailed questions about the perception of added value. For this purpose, future studies should focus on the perceived added value of PD expertise in a more extensive way.

Second, we do not know whether the participants’ overestimation of the expertise of their physiotherapist correlates with their satisfaction with their treatment and physiotherapist in general. We did not take the element of consumer satisfaction into account. Future work should replicate these findings and control for consumer satisfaction when asking about physiotherapist expertise in PD. A recent paper shows that consumers are generally very satisfied with their physiotherapeutic care [31].

Third, only a minority searched for CPI so that the influence of the variables could not always be estimated precisely, which the large confidence intervals reflect.

Fourth, information about the number of physiotherapy episodes respondents had in the past year would brought us more insight whether the choice for a physiotherapist was temporally or more on and on. Future research should use items about the number of episodes, the length of the episodes and number physiotherapists they were treated during these episodes.

A strength is that our sample is representative: the mean age of 72 ± 9 years is in line with the data based on the Dutch system of diagnosis-treatment combination (71 ± 10 years). The gender proportion (56% men) was also consistent with the national number [32]. According to the Dutch Parkinson guideline, there are no data available regarding the severity of the disease.

## Conclusions

This study shows that recognition of the added value of an expert physiotherapist was found to be the strongest predictor for the search for CPI. The definition of expertise expressed by PwP was in line with patient-centeredness, though in a rather narrow manner, as only certain characteristics of PD expertise were recognised. In order for PwP choosing high-quality care, improvements are needed. There is a lack of recognition of expertise caused by a mismatch in the current available CPI. As PwP showed relatively few signs of selectively choosing expert physiotherapists, CPI should include additional and crucial quality of care information that matters for PwP. After this, it is expected that expert physiotherapists become more easier recognisable for those who want to make a selective provider choice. Furthermore, PwP heavily rely on their referring providers, meaning that referring providers have a responsibility to act as a coach for their patients. This fact should be used advantageously by involving the professionals in a more active way.

## Abbreviations

CI: Confidence interval; CPI: Comparative performance information; GP: General practitioner; HY: Hoehn and Yahr; OR: Odds ratio; PD: Parkinson’s disease; SD: Standard deviation.

## Competing interests

The authors declare that they have no competing interests.

## Authors’ contributions

NK, MM, and MF conceived the study and the design for the survey. NK analysed and interpreted the data and wrote a draft manuscript. MM and MF critically revised the manuscript. All authors read and approved the final manuscript.

## Pre-publication history

The pre-publication history for this paper can be accessed here:

http://www.biomedcentral.com/1472-6963/13/430/prepub
